# Patterns in bottlenecks for implementation of health promotion interventions: a cross-sectional observational study on intervention-context interactions in the Netherlands

**DOI:** 10.1186/s13690-023-01196-y

**Published:** 2023-10-17

**Authors:** K. M. Grêaux, P. van Assema, K. M. H. H. Bessems, N. K. de Vries, J. Harting

**Affiliations:** 1 Department of Health Promotion, NUTRIM School of Nutrition and Translational Research in Metabolism, Maastricht University Medical Centre+, PO Box 616, 6200 MD Maastricht, the Netherlands; 2 Caphri School of Public Health and Primary Care, Department of Health Promotion, Maastricht University Medical Centre+, PO Box 616, Maastricht, 6200 MD the Netherlands; 3 Department of Public and Occupational Health, Amsterdam UMC, University of Amsterdam, Meibergdreef 9, Amsterdam, the Netherlands; 4 Amsterdam Public Health Research Institute, Health Behaviors and Chronic Diseases Research Programme, Amsterdam, the Netherlands

**Keywords:** Implementation, Health promotion, Intervention-context interactions, Bottlenecks

## Abstract

**Background:**

From a complex systems perspective, implementation should be understood as the introduction of an intervention in a context with which it needs to interact in order to achieve its function in terms of improved health. The presence of intervention-context interactions could mean that during implementation particular patterns of crucial interaction points might arise. We examined the presence of – and regularities in – such ‘bottlenecks for implementation’, as this could create opportunities to predict and intervene in potential implementation problems.

**Methods:**

We conducted a cross-sectional observational study against the background of municipal intersectoral policymaking in the Netherlands. We asked implementers of health promotion interventions to identify bottlenecks by rating the presence and importance of conditions for implementation in a range of intervention systems. We used descriptive statistics to characterize these systems (by their behaviour change method, health theme and implementation setting) and the conditions that acted as bottlenecks. After stratifying bottlenecks by intervention system and the system’s characteristics, we tested our hypotheses by comparing the number and nature of the bottlenecks that emerged.

**Results:**

More than half of the possible conditions were identified as a bottleneck for implementation. Bottlenecks occurred in all categories of conditions, e.g., relating to the implementer, the intervention, and political and administrative support, and often connected with intersectoral policymaking, e.g., relating to the co-implementer and the co-implementer’s organization. Both our hypotheses were supported: (1) Each intervention system came across a unique set of – a limited number of – conditions hampering implementation; (2) Most bottlenecks were associated with the characteristics of the system in which they occurred, but bottlenecks also appeared in the absence of such an association, or remained absent in the presence thereof.

**Conclusions:**

We conclude that intervention-context interactions in integrated health policymaking may lead to both regularities and variations in bottlenecks for implementation. Regularities may partly be predicted by the function of an intervention system, and may serve as the basis for building the capacity needed for the structural changes that can bring about long-lasting health improvements. Variations may point at the need for flexibility in further tailoring the implementation approach to the – mostly unpredictable – problems at individual sites.

**Supplementary Information:**

The online version contains supplementary material available at 10.1186/s13690-023-01196-y.

**Table:** 

Text box 1. Contributions to the literature	
• Findings of this study indicate how the implementation of health promotion interventions may be improved by identifying a minimized list of bottlenecks for implementation to be focused on. • These findings contribute to the existing knowledge gap on intervention-context interactions, by asserting that more tailored implementation strategies can be created trough early predictions of potential bottlenecks based on the characteristics of the intervention and the implementation setting. • Although we found evidence for early predictions of potential bottlenecks, the results also imply that any implementation plan needs to be flexible enough to further tailor implementation approaches to unpredictable problems that can occur at individual sites.	

## Introduction

### Background

Intersectoral health policy is an important approach to improving public health [1–3]. It usually includes the implementation of health promotion interventions that employ several behaviour-change methods to address multiple health themes in a variety of local settings [4–6]. From a complex systems perspective, this implementation should be understood as the introduction of an intervention in a context with which it needs to interact [7]. It is through this interaction that an intervention becomes adopted [8], changes individual behaviours and builds the capacity to achieve an intervention’s ‘function’ in terms of long-lasting health improvements [7, 9–11]. Conceiving implementation as an intervention-context interaction implies that the implementation setting serves as an active intervention element, rather than as an inactive site offering access to a population and/or a space to carry out an intervention as it is [7, 12].

The presence of intervention-context interactions could mean that during implementation, depending on the nature of both the intervention and the context, particular patterns of interaction points might arise [7, 9]. Such a pattern would then reflect the crucial areas where a specific intervention has to combine with a particular context to perform its function [7, 9, 13, 14]. Such an interaction pattern was, for instance, found in a multiple case study that observed how the introduction of a social-emotional learning intervention in schools ran into comparable problems across different school settings, e.g., with respect to ensuring the intervention’s congruence with contextual needs and resources [15]. These implementation problems were interpreted as unfavourable interactions between specific intervention characteristics and typical features of the setting, requiring either adjustment of the intervention, or capacity building in the implementation setting, or transformations of both the intervention and the context [15]. Identifying patterns in such key intervention-context interaction points, which we call ‘bottlenecks for implementation’, could create opportunities to predict and intervene in potential implementation problems [7, 15].

As empirical studies on intervention-context interactions are considered important but scarce [8], we examined the presence of – and patterns in – such interactions against the background of municipal intersectoral health policymaking in the Netherlands. This background offered the unique opportunity to include, as recommended [8], a diversity of health promotion interventions in a variety of local contexts in our study. In this manuscript, we will describe and compare the bottlenecks for implementation that occurred in different ‘intervention systems’ [10, 14]. Such an intervention system includes both the interventional components (i.e. the behaviour change method used and the health theme addressed) and the contextual elements (i.e. the implementation setting) [10]. We regard these components and elements as the core characteristics of an intervention’s causal theory that reflects the function of an intervention in terms of its health promoting effects [10, 13].

### Hypothesis 1

Individual empirical studies provide two different indications for the presence of regularities in intervention-context interactions. The first is that in similar intervention systems, that have comparable intervention components and contextual elements, identical sets of bottlenecks for implementation are likely to arise. For example, one multiple case study observed that the implementation of health promotion programmes in schools was hampered by recurrent combinations of a limited number of contextual factors, such as the support from the municipality and the involvement of the community [16]. Another multiple case study found that just some of all possible conditions for implementation, such as the formal ratification by the management, actually hampered the introduction of an intersectoral approach targeting childhood obesity in local communities [17].

The second indication of regularities in intervention-context interactions is that in dissimilar intervention systems different sets of bottlenecks for implementation tend to emerge. For example, a cross-sectional survey on the introduction of prevention programmes in schools found that partly different factors were involved in the implementation of individual-level programs targeting student behaviour, such as the characteristics of the programme and the school, than in that of environmental-level programmes addressing the school climate, such as the support from the school principle and the organizational capacity [18, 19].

Together, these indications for the presence of patterns in intervention-context interactions led to our first study hypothesis (H-1), stating that each distinct intervention system will encounter a unique set of bottlenecks during implementation.

### Hypothesis 2

In general, reviews of implementation studies do not result in a limited set of factors that would similarly influence implementation in an intervention system. Instead, such reviews typically identified ‘hundreds’ of different influential factors [8, 20], of which many were found to alternatively facilitate and hamper implementation in a particular intervention system [8, 20]. Examples of factors with such a dual role were the contextual appropriateness of school-based physical activity programmes for healthy youth [21], the collaboration between community partners in intersectoral approaches targeting child obesity [22], and a multicomponent approach in home injury prevention programmes for pre-school children [23]. Findings like these point at the presence of context-dependency in intervention-context interactions [20].

Therefore, next to expecting regularities in bottlenecks in a certain intervention system, bottlenecks should also be assumed to vary within such a system [20]. This assumption was supported by empirical studies that, next to regularities, found variations in the conditions for implementation within a particular intervention system [16, 18, 19]. For example, despite a recurrent combination of a small number of relevant conditions across schools (see above), at the level of individual schools, the influential factors, such as the availability of staff and the cohesion of the school team, appeared to be highly specific and variable [16]. Hence, the characteristics of an intervention system, i.e. its behaviour change method, health theme and implementation setting, might be both essential in themselves *and* have to interact in order to allow an intervention to realise its intended function [20, 24].

Together, our second study hypothesis (H-2) reads that bottlenecks for implementation (H-2a) are partly associated with the specific characteristics of a particular intervention system (due to the essentiality of these characteristics) *and* (H-2b) will partly arise independent of these characteristics (due to their mutual interaction).

## Methods

### Design

We examined intervention-context interactions in a cross-sectional observational study (2012–2014). Included were 30 municipalities or alliances of municipalities participating in a ministerial programme on intersectoral health policymaking. Four other projects in this programme were not eligible: one prematurely ended its participation in the programme, one did not implement interventions in the years concerned, and two refused permission to approach the partners responsible for the implementation of the interventions.

### Study setting

The ministerial programme (2009–2015) was initiated by the Dutch Ministry of Health, Welfare and Sport . The programme gave municipalities the opportunity to experiment with intersectoral health policymaking over a period of 24–48 months. Municipalities or alliances thereof could apply for participation in the programme. One requirement was the appointment of a project leader who had to adopt a coordinating role in establishing local partnerships and implementing health promotion interventions. The employment of the project leader was covered by the financial support provided by the ministerial programme. This financial support also partly covered the implementation of the health promotion interventions. The ministerial programme additionally provided professional support addressing, for instance, the selection and implementation of evidence-based health promotion interventions.

As previously reported [25], the local partnerships in the projects encompassed an average of seven different sectors (e.g., public health, education and transportation). The health promotion interventions applied a variety of behaviour change methods (e.g., education, facilitation and regulation), to address overweight, alcohol use (sometimes in combination with drugs and smoking) or other health themes, in a range of local settings (e.g., school settings and outdoor public sites). The intervention-context combinations that most often were being implemented in the projects are characterized in Supplementary file 1.

The implementation of interventions was mostly carried out by one of the partners in the project (i.e. the implementer) and supported by one or more other partner organizations (i.e. co-implementers working at co-implementing organizations). Most of the implementers worked for a municipal government organization, and almost half of them for a health organization. On average, the implementers had 10 years of relevant work experience.

### Data collection

Details about the data collection have been reported elsewhere [25]. In brief, the data was collected from 2012 to 2014 (inclusive). Two questionnaires were used: one for project leaders (*n* = 30) and one for implementers of the interventions (*n* = 181). For the present study, both the project leaders and the implementers were asked to complete questions regarding the characteristics of the intervention systems (*n* = 424). The implementers had to complete additional questions about the conditions acting as bottlenecks for implementation.

#### Intervention system

##### *Questionnaire items*

The project leaders were asked to report the names of the health promotion interventions being implemented in their project. The implementers were asked, for each intervention they were responsible for, to concisely describe its aim, topic, content/components and implementation setting.

##### *Data processing*

We operationalized the intervention system using three proxy measures for its function: the core behaviour change method employed, the main health theme addressed, and the primary setting of implementation [10, 26]. The core method of behaviour change was retrieved from the aim and content of the health promotion intervention, and categorized into [6]: education (e.g., school learning module), regulation (e.g., legislation regarding the sale of alcohol products in sports ground cafeterias), facilitation (e.g., environmental changes, such as new play gardens), citizen participation (e.g., citizens organizing a walking event), and case finding (e.g., spotting drunk youngsters in nightlife). The main health theme was inferred from the topic, aim and content of the intervention, and categorized into overweight (e.g., nutrition and physical activity), alcohol (sometimes in combination with drugs and smoking) and other health themes (e.g., fall prevention or self-defence). The primary implementation setting was derived from the description by the prime implementer, and categorized into [4]: schools or preschools, outdoor public sites (e.g., playgrounds, nature areas), sports facilities, homes (including websites to be consulted at home), commercial buildings (e.g., supermarkets, bars, restaurants), health or welfare buildings (e.g., hospitals, welfare organizations, addiction centres), and public buildings (e.g., libraries, community centres).

#### Bottlenecks for implementation

##### *Selecting conditions*

An extensive review of the literature resulted in a list of 125 conditions necessary for the implementation of health promotion interventions in local settings [8, 27–32]. To select the conditions relevant to our study, we held 17 semi-structured telephone interviews: five with Dutch implementation experts and twelve with Dutch health promotion professionals responsible for local implementation. None of the interviewees was participating in the ministerial programme. Guided by an implementation framework [27], but without being provided with the prepared list, they were asked to name those conditions that were most important in the context of intersectoral policymaking. The 47 conditions that were mentioned most were included in the questionnaire for the prime implementers.

##### *Questionnaire*

The relevant conditions were organized into seven categories (i-vii) [27], that we adapted to the context of intersectoral policymaking, e.g., by referring to an integrated approach, and by making separate categories of conditions for the co-implementer(s) and co-implementing organization(s). Conditions were framed as statements: (i) five related to the prime implementer (e.g., ‘I have sufficient skills to implement the intervention’); (ii) five to the co-implementer(s) (e.g., ‘Other professionals are capable enough to implement the intervention’); (iii) ten to the intervention (e.g., ‘The intervention is easy to carry out’); (iv) ten to the prime implementer’s organization (e.g., ‘The intervention fits my organization’s policy’); (v) eleven to co-implementer’s organization(s) (e.g., ‘Other organizations sufficiently support the intervention’s health theme’); (vi) four to the broader context (e.g., ‘There is enough administrative and political support for the intervention’); and (vii) two to the implementation strategy employed (e.g. ‘Good materials required for implementation are available’). For the complete questionnaire, see Supplementary file 2.

To assess the extent to which the conditions for the implementation of the intervention under consideration were regarded as being present, the prime implementers had to score each statement on a five-point scale (from strongly disagree to strongly agree). To assess the perceived importance of the conditions, the prime implementers were asked to select the five conditions they regarded as most important for the successful implementation of the intervention. We opted for this top-5 of importance as to discriminate the expected limited number of crucial conditions [16, 17] from the myriad of potential conditions for implementation [8, 20]. For their top-5, the implementers could refer to the 47 conditions in the list or add a condition not included in the list. Of the added conditions, half could be recoded as a prelisted condition. The other half, making up 11% of all answers, were not specific enough to be categorized (e.g., a lack of time, insufficient skills or short of manpower in general), and were not further taken into account.

##### *Data processing*

For each individual intervention, the perceived presence of each of the conditions for implementation was dichotomized into being ‘optimal’ if a prime implementer indicated strong agreement with the corresponding statement, and being ‘sub-optimal’ for all alternative answers. This cut-off point was chosen because of the skewed distribution of perceived presence: any other division would have minimized the percentage of interventions for which a condition was marked as ‘sub-optimal’, leaving many bottlenecks undetected. Next, conditions were marked as ‘important’ if assigned to the top 5, irrespective of their position therein. Finally, conditions were marked as a bottleneck if they were perceived as being both ‘important’ and ‘sub-optimal’.

##### *Data analysis*

Descriptive statistics were used to characterize the included intervention systems, and to calculate the percentage of systems in which a condition for implementation was marked as sub-optimal, important and a bottleneck.

We tested our study hypotheses using stratified analyses (see below). To warrant the availability of sufficient observations for hypotheses testing, we selected the intervention systems that were most frequently present in our sample (*n* > 10; see Supplementary file 1). After stratification, a condition was regarded a bottleneck for implementation if it was marked as such in more than 10% of the intervention systems that made up a certain stratum, i.e. a certain subset of systems.

To test our first hypothesis (H-1), we stratified the percentage of bottlenecks by frequent intervention system. To assess whether each distinct intervention system came across a unique set of bottlenecks for implementation, we compared the number and the nature of the conditions that emerged as bottlenecks in the different strata, i.e. in the different subsets of intervention systems. All comparisons were observational.

To test our second hypothesis (H-2), we additionally stratified the percentage of bottlenecks by intervention system characteristics: the behaviour change method, health theme and implementation setting. We then compared the conditions that were regarded a bottleneck after each of the stratification procedures. To indicate that a bottleneck was associated with the characteristics of a particular intervention system (H-2a), we labelled it ‘expectedly present’ in that system if the condition involved also acted as a bottleneck in all systems having a characteristic in common. To indicate that a bottleneck emerged independent of the characteristics of a particular intervention system (H-2b), we labelled it ‘unexpectedly present’ in that system if the condition involved did not act as a bottleneck in all systems having a characteristic in common. In addition, a bottleneck was labelled ‘unexpectedly absent’ if the invers incongruence was true, i.e. if a condition did not act as bottleneck in a particular system, while it did so in all intervention systems having a characteristic in common.

## Results

### Response

A total of 120 implementers (response rate 66.3%) provided data about 243 intervention systems (response rate 57.3%) implemented in 30 projects. Response details are shown in Supplementary file 3.

### Intervention systems

In all intervention systems, education was the most frequently used core method of behaviour change  (*n* = 137; Table 1, Behaviour change method columns). Less often applied were facilitation (*n* = 57), regulation (*n* = 25), case finding (*n* = 13) and citizen participation (*n* = 11). Overweight was the most frequently addressed health theme in the intervention systems (*n* = 123; Health theme columns). Alcohol (*n* = 102) and other health themes (*n* = 16) were addressed less often. The school setting (*n* = 75; Implementation setting columns) most often served as the primary implementation setting. Less frequently used were outdoor public sites (*n* = 38), public buildings (*n* = 38), health or welfare buildings (*n* = 24) sports facilities (*n* = 24), commercial buildings (*n* = 24) and the home setting (*n* = 15).

**Table 1:**
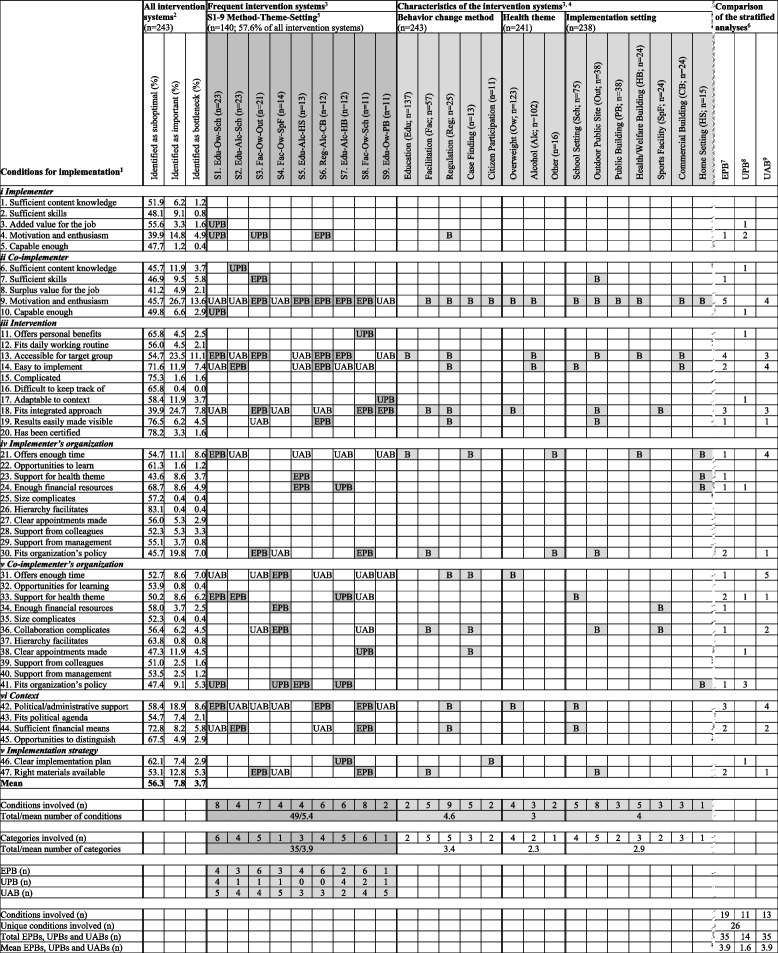
Implementation bottlenecks: all intervention systems, and stratified by intervention system and its characteristics

^**1**^ The questionnaire including the full statements on the conditions for implementation is available in Supplementary file 2

^2^ There was a negative correlation between identified as suboptimal and conditions identified as important: Pearson’s *r* = -.40; *p* = .005

^3^  = Shaded cells including one or more characters (B, EPB or UPB) indicate the presence of a bottleneck;  = Unshaded cells that are empty or include UAB indicate the absence of a bottleneck

^4^ B = Bottleneck

^5^ S1-S9 = Frequent intervention systems (characterized in Supplementary file 1)

^6^ Comparison of the stratified analyses: bottlenecks being present in a frequent intervention system compared to those present in all systems sharing one of the frequent system’s characteristics

^7^ EPB = ‘Expectedly present bottleneck’ (i.e. present both for in a frequent intervention system and in all intervention systems sharing one of the frequent system’s characteristics)

^8^ UPB = ‘Unexpectedly present bottleneck’ (i.e. present for in a frequent intervention system, but not in all interventions that share one of the frequent system’s characteristics)

^9 ^UAB = ‘Unexpectedly absent bottleneck’ (i.e. not present in an intervention system, but present in all interventions sharing one of the frequent system’s characteristics)

Nine intervention systems were present more than ten times. Together, these nine frequently present systems covered 140 of all 243 systems in the sample (58%; Table 1, S1-9 Method-Theme-Setting columns; see also Supplementary file 1). In five of the frequent intervention systems, the core behaviour change method applied was education. Facilitation was used in three, and regulation in one of the frequently present systems. In five of these systems, the main theme addressed was overweight, and in the other four this was alcohol. Schools were the primary implementation setting in three of the frequent intervention systems. The other six such systems included a different setting each.

### Conditions

On average, conditions were considered to be sub-optimally present in 56.3% of all intervention systems (range 39.9–83.1%; Table 1; All intervention systems columns), and placed in the top 5 of importance in 7.8% of the intervention systems (range 0.4–26.7%). Conditions were regarded to be both sub-optimally present and of great importance, i.e. as a bottleneck for implementation, in 3.7% of all intervention systems (range 0–13.6%). For further details, see Supplementary file 4.

### Bottlenecks

#### General observations

In total, in the frequent intervention systems, 26 conditions (55.3% of all possible conditions; Table 1, Comparison of the stratified analyses columns) were at least once perceived to act as bottlenecks for implementation, while the other 21 conditions were never identified as such. Two conditions were identified as bottlenecks in more than 10% of all intervention systems: the motivation and enthusiasm of the co-implementer(s) (13.6%) and the accessibility of the intervention for the target group (11.1%; Table 1, All intervention systems columns). These two conditions hampered implementation in five and four frequent intervention systems, respectively. Although less often identified as bottlenecks in all systems (4.9%-8.6%), one other condition acted as such in four frequent intervention systems, i.e. whether the intervention fitted the policy of the co-implementer’s organization, and four others did so in three such systems, i.e. the motivation and enthusiasm of the implementer, whether the intervention fitted an integrated approach, the support for the health theme in the co-implementer’s organization, and the contextual political or administrative support.

The other 19 conditions that were identified as a bottleneck at least once, acted as such in one or two of the frequent intervention systems. Eight of these conditions were found to hamper implementation in all systems relatively frequent (4.9%-8.6%). Half of these conditions referred to being offered enough time (i.e. by the implementer’s and the co-implementer’s organizations) and to the presence of sufficient financial means (i.e. in the implementer’s organization and in the broader context). The other half included the skills of the co-implementer, if the intervention was easy to implement, if the intervention fitted the policy of the implementer’s organisation, and having the right materials available for the implementation strategy.

##### ***H-1****: The number and nature of bottlenecks depend on the intervention system*

The conditions perceived to be a bottleneck differed in the frequent intervention systems regarding both their number and nature (Table 1, S1-9 Method-Theme Setting columns). The average number of conditions identified as a bottleneck was 5.4, with a range of two to eight per intervention system. On average, these bottlenecks represented 3.9 categories of conditions, with a range of one to six categories per intervention system. For example, intervention system S1, in which education was used to address overweight in schools, was associated with eight bottlenecks in six categories, i.e. in all but that of the implementation strategy. Another example is S9, where education was used to address overweight in public buildings. This system was associated with two bottlenecks in one category, i.e. the characteristics of the intervention.

In terms of their nature, each intervention system had its own set of bottlenecks for implementation. Although every random pair of intervention systems had at least one bottleneck in common, in each individual system implementation was hampered by at least one unique condition, i.e. one that did not act as a bottleneck in any other system. For example, S4, in which facilitation was used to address overweight in sports facilities, shared one bottleneck with three other systems (S1-5–7), i.e. whether the intervention fitted the policy of the co-implementer’s organization. However, implementation in S4 was additionally hampered by three unique bottlenecks that were also related to the co-implementer’s organisation, e.g., complications because of interorganizational collaboration. The maximum number of bottlenecks that one pair of frequent intervention systems had in common was four. These were S3 and S8, in which facilitation was used to address overweight, in outdoor public sites and schools, respectively. Three common bottlenecks were whether the intervention fitted an integrated approach and the policy of the implementer’s organization, and if the right materials for the implementation strategy were available. One unique bottleneck in S3 were the co-implementer’s skills.

Different sets of bottlenecks were identified if a similar method was applied to address the same health theme, but in a different setting. For instance, in S3 and S4, where facilitation was applied to address overweight in different settings, bottlenecks for implementation emerged in different categories of conditions. The bottlenecks in outdoor public places were related to the implementer, the co-implementer and the intervention, and in sports facilities to the co-implementer’s organization. Another example includes S2 and S7, in which education was used to address alcohol in schools and health or welfare buildings, respectively. The only bottleneck for implementation that these systems had in common was the support for the health theme in the co-implementer’s organization. This condition was also the only common bottleneck for implementation in S1 and S2, where education was used in the school setting, to address overweight and alcohol, respectively. This illustrates that the nature of bottlenecks also could differ in intervention systems where a similar behaviour change method was used in a comparable setting, but to address a different health theme.

##### ***H-2a. Bottlenecks are associated with the characteristics of an intervention system***

Of all 49 bottlenecks identified for the frequent intervention systems (Table 1; S1-9 Method-Theme-Setting columns), 35 were ‘expectedly present’ (EPBs; 71.4% of all bottlenecks; Comparison of the stratified analysis columns). This means that the majority of the bottlenecks was associated with the characteristics of the intervention system. In both S5 and S6, only EPBs emerged. The conditions acting as bottlenecks in S5, in which education was used to address alcohol in the home setting, were also identified as bottlenecks in other intervention systems with the home setting. One of these bottlenecks was the financial means available from the implementer’s organization. The conditions acting as bottlenecks in S6, which involved the regulation of alcohol in commercial buildings, were also identified as bottlenecks for other interventions applying regulation as a method. These conditions included, among others, whether the intervention easily could be implemented. The mean number of EPBs per frequent intervention system was 3.9 (range 1–6).

##### ***H-2b****: Bottlenecks arise independent of the characteristics of an intervention system*

The other 14 bottlenecks identified for implementation in the frequent intervention systems were ‘unexpectedly present’ (UPBs; 28.6% of all bottlenecks; Table 1; Comparison of the stratified analyses columns). This means that a minority of the bottlenecks emerged independent of the characteristics of the intervention system. About one third of the UPBs concerned conditions that were not identified as a bottleneck in any of the other stratified analyses. For instance, in S9, in which education was used to address overweight in public building, implementation was unexpectedly hampered by the adaptability of the intervention to the context, a condition that was not associated with any of the system’s characteristics. The other two thirds of the UPBs involved conditions associated with one or more characteristics, but not with those of the intervention system itself. For example, in S8, in which facilitation was used to address overweight in schools, implementation was unexpectedly hampered by the clarity of the appointments made with the co-implementer’s organization, a condition that in the other stratified analyses was associated with case finding as a method. Most conditions identified to act as an UPB did so in only one of the frequent intervention systems. One exception was the motivation and enthusiasm of the implementer, that emerged as an UPB in both S1, where education was used to address overweight in schools, and S3, where facilitation as applied on overweight in outdoor public sites. The average number of UPBs per intervention system was 1.6 (range 0–4).

For implementation in the frequent intervention systems, 35 bottlenecks were ‘unexpectedly absent’ (UABs). This once more indicates that bottlenecks for implementation emerged independent of the characteristics of the intervention system. In S9, for example, in which education was used to address overweight in public buildings, five conditions were not identified as a bottleneck, while they did emerge as such after stratification by the characteristics of the intervention system. The UABs in S9 also included the two conditions most frequently perceived to be a bottleneck, i.e. the motivation and enthusiasm of the co-implementer and the accessibility of the intervention for the target group. Other conditions regularly identified as UABs included the available time from both the implementer’s and the co-implementer’s organization, as well as the contextual political and administrative support. The mean number of UABs per frequent intervention system was 3.9 (range 2–5).

## Discussion

### Summary of the findings

This cross-sectional observational study examined patterns in problematic intervention-context interactions – i.e. bottlenecks for implementation – during the introduction of health promotion interventions as part of local intersectoral health policymaking in the Netherlands. Of the possible conditions for implementation, more than half acted as a bottleneck at least once, while less than a half were never identified as such. Bottlenecks were found in all categories of conditions, e.g., those relating to the implementer, the intervention, and political and administrative support, and often connected with the intersectoral policymaking, e.g., those relating to the co-implementer and the co-implementer’s organization. Our stratification procedures supported both our hypotheses. In agreement with our first hypothesis, each distinct intervention system, i.e. each particular combination of behaviour change method, health theme and local setting, came across a unique set of – a limited number of – conditions hampering implementation. Regarding the first part of our second hypothesis, we found that the bottlenecks for implementation in a particular system were more often than not associated with the system’s characteristics representing its function in terms of its health promoting effects. Regarding the second part of that hypothesis, we saw – to a lesser extent – that conditions for implementation served as a bottleneck in a particular system independent of the system’s characteristics, or – to a greater extent – did not act as a bottleneck despite the presence of such an association.

### Interpretation

Our study provides twofold support for the complex systems perspective which says that during implementation, interventions interact with the context in which they are being introduced [7, 9, 14]. First, our results support the assumed presence of patterns in these interactions [7, 15]. The regularities we found in the conditions that acted as bottlenecks for implementation can possibly be explained by the way structural factors, i.e. the socio-economic and political context, are arranged, and which are operating ‘one level up’ from an intervention [33, 34]. These – often given and fixed – factors in the wider context [20] may more or less similarly shape the more flexible conditions of comparable local sites [10, 14, 33]. For example, the observation that the bottlenecks for implementation in our study were often related to intersectoral policy making, e.g., to the integrated approach, co-implementer or co-implementing organization, could indicate a shaping role of the – at that time – sectoral national policy landscape [35]. That is, such a sectoral national policy might explain the bottlenecks we observed in the broader political and administrative support as well as those in the co-implementer’s organization, like the support for the health theme and whether an intervention fitted such a co-organization’s policy.

Regularities in bottlenecks could create opportunities to predict and intervene in potential implementation problems [7, 15]. Our study supports the idea that the function of an intervention, in terms of the characteristics that reflect its causal theory, could be a helpful starting point for an early identification of – probably a limited number of – bottlenecks [7, 10]. In view of the above-discussed role in shaping the conditions of local settings, it might be worthwhile to direct such an early assessment at structural factors, and to prioritize these in designing implementation plans [18, 20, 33]. For example, the bottlenecks that our study found in the intervention system in which regulation was used to address alcohol in local commercial buildings, might reflect the permissive cultural norm towards the consumption of alcohol in the Netherlands [36]. Such structural factors, i.e. those that constitute and tend to preserve the complex system in which interventions are being introduced [7], may be effectively changed by nation-wide strategies, such as advocacy, laws and regulations [6]. Hence, it might require strategies like these to build the capacity needed to bring about the comprehensive and long-lasting health improvements that most previous programmes have so far failed to achieve [7, 11].

The second type of support for the systems-based perspective is that the bottlenecks for implementation in our study seemed to be produced by, or disappear through, intervention-context interactions [7, 8]. This means that not all bottlenecks for implementation can be predicted from the function of an intervention: some may be unexpectedly present, others may be unexpectedly absent. This is in agreement with previous studies which, despite the presence of regularities, found a great variation in conditions hampering the introduction of similar interventions at identical implementation sites [16, 20]. One explanation for this variation could be that local implementation sites that make up one type of setting may still differ importantly in a number of features [37]. That is, despite the same structural factors, such as a sectoral national policy landscape [35], the actual implementation sites may vary substantially in their local response. This can be due to differences in local factors [14, 20, 33], like the degree to which a municipal policy approach is intersectoral [38]. In our study, such a variation in local responses may be illustrated by the bottlenecks for implementation that either were unexpectedly present, e.g., the motivation and enthusiasm of the implementer, or unexpectedly absent, e.g., whether the intervention fitted an integrated approach. In other words, the individual make-up of implementation sites may – through different intervention-context interactions – create unpredictable variations in the bottlenecks for implementation. This means that any initial implementation plan, including strategies aimed at changing structural factors, should be flexible to allow further local tailoring to individual sites.

### Strength and limitations

We were able to analyse patterns in bottlenecks for implementation, because our study included a large number and a wide variety of health promotion interventions in a broad range of settings. This allowed us to quantitatively compute and qualitatively compare these bottlenecks in no less than nine different intervention systems. In doing so, our study may serve as an example of how the impact of context on implementation might be more systematically studied [8]. Additional in-depth understanding of intervention-context interactions might come from social network studies, actor network studies or realist approaches [10, 14, 39].

A drawback of the wide variety of interventions was that the nine frequent intervention systems that were central to our analyses covered no more than 58% of all systems included in our study. Also, these nine systems represented just 21% of all method-theme-setting combinations in our study, and less than 9% of all possible combinations. Underrepresented or absent in our analyses were interventions applying regulation, citizen participation or case finding; overrepresented were interventions using education or facilitation. This distribution of behaviour change methods may reflect a common tendency in health promotion to use interventions that at best minimally disrupt the context in which they are being introduced [13]. As a consequence, our study was not able to identify bottlenecks for interventions that aim to bring about more structural changes, though our findings suggest that these bottlenecks would at least partly differ from the ones we observed.

Another strength is that our study started from the – essential – function of an intervention, rather than from its – adaptable – form or components [7, 24]. A limitation could be that ‘function’ was rather pragmatically operationalized: we used proxies that we could infer from the available information and that we expected to reflect the intervention’s theory of change [7, 10]. Though these proxies enabled us to examine intervention-context interactions – or bottlenecks for implementation – in different intervention systems, their selection (e.g., the core behaviour change method rather than the mix of such methods) and the high level of aggregation could also be criticized. Future studies might wish to experiment with using a more finely grained taxonomy of behaviour change methods [40], specifying sub-categories within aggregated types of settings [37], adding the target group or the health behaviour determinants addressed [6], or using a more general approach, like a community or intersectoral approach, as the level of analysis [13, 41]. For instance, the use of a more general approach could better reflect the mix of behaviour change methods that is recommended to improve health [13], and thus contribute to the identification of the bottlenecks associated with the implementation thereof.

A final strength is that our implementers both assessed the degree to which conditions for implementation were present and selected the ones that they regarded as the most important. Here, a first limitation could be that we labelled a condition a ‘bottleneck’ for implementation if the implementers had scored it as both sub-optimal and of great importance for a minimum of 10% of the interventions included in the analysis. Although this 10% cut-off point may seem low, our definition of ‘very important’, i.e. the implementer placed a condition in the top 5 of importance, was already very restrictive. In doing so, we aimed to select only ‘real’ bottlenecks, which may be assumed to encompass only a small number of the multitude of potential hampering conditions [16, 17]. A second limitation could be our definition of ‘sub-optimal’, i.e. the implementer did not strongly agree that a condition was present. This definition implied that the other, in part equivocal perceptions of presence (i.e. strongly disagree up to and including agree) were merged and classified as ‘not sub-optimal’. However, this categorization appeared to be necessary, as the skewed distribution of perceived presence would otherwise have left many bottlenecks undetected. Taken together, we believe that our approach was both sufficient selective and sensitive enough to identify the relevant bottlenecks for implementation in intervention systems. However, studies using our cut-off points and definitions might either underestimate or overestimate the real number of bottlenecks in health promotion practice.

## Conclusion

Starting from a complex systems perspective on implementation, our findings support the presence of intervention-context interactions. These interactions may produce both regularities and variations in bottlenecks for implementation. Regularities may serve as the – partly predictable – basis for implementation strategies aimed at building the capacity needed for the structural changes that can bring about long-lasting health improvements. Variations in bottlenecks may point at the need for flexibility to tailor implementation approaches to the – mostly unpredictable – implementation problems at individual sites.

### Supplementary Information

**Additional file 1:** **Supplementary file 1.** Characterization of the most frequently present intervention systems.

**Additional file 2:** **Supplementary file 2.** Questionnaire for the survey on conditions for implementation of interventions.

**Additional file 3:** **Supplementary file 3.** Flowchart of the response to the survey on conditions for implementation of interventions.

**Additional file 4:** **Supplementary file 4.** Conditions identified as suboptimal, important and bottleneck, both for implementation in all intervention systems, and stratified by frequent intervention system and by characteristics representing the funtion of the systems.

## Data Availability

The datasets used and analysed during the current study are available from the corresponding author on reasonable request.

## Abbreviations

EPB: Expectedly present bottleneck
UPB: Unexpectedly present bottleneck
UAB: Unexpectedly absent bottleneck
