# Supplementary material for: Patterns in bottlenecks for implementation of health promotion interventions: a cross-sectional observational study on intervention-context interactions in the Netherlands
Source: Arch Public Health. 2023 Oct 17;81:183. doi: 10.1186/s13690-023-01196-y (PMC10580618; doi:10.1186/s13690-023-01196-y)
Supplement: Supplementary file 1 — Additional file 1: Supplementary file 1. Characterization of the most frequently present intervention systems. [file 13690_2023_1196_MOESM1_ESM.docx]

**Supplementary file 1. Characterization of the most frequently present intervention systems**

| **Intervention systems** | **Description** |
| --- | --- |
| S1. Education-Overweight-School Setting | Interventions providing knowledge, creating awareness, or teaching how to make healthy decisions regarding nutrition and/or physical activity. The interventions were mainly implemented in primary and secondary schools. They mostly comprised educational materials and instructions for a cohesive series of teacher-led lessons. They sometimes also included smaller, once-only and easily accessible information sessions, training sessions or meetings for parents at the school. |
| S2. Education-Alcohol-School setting | Interventions either providing knowledge and creating awareness about the risks of alcohol, drugs and smoking, or building resilience against alcohol, drugs and smoking. The interventions were mainly implemented in primary and secondary schools. They mostly consisted of educational materials and instructions for one or more teacher-led sessions. They sometimes also included smaller, once-only and easily accessible information sessions, training sessions or meetings for parents at the school. |
| S3. Facilitation-Overweight-Outdoor Public Sites | Interventions typically included organizing incidental, small-scale and easily accessible physical activity opportunities in outdoor public spaces (e.g., walking, running or outdoor game activities in the neighbourhood). They primarily promoted physical activity or tried to show that being physically active is fun. The interventions sometimes also included longer-lasting physical adjustments (e.g., creating playgrounds or providing sports materials or healthy nutritional products in parks/recreation areas). |
| S4. Facilitation-Overweight-Sports Facilities | Interventions mostly comprised organizing incidental, small-scale and easily accessible sports activities at sports facilities. The main aim was to motivate people to be more physically active or create awareness of the existence of – and promote participation in – a specific sport (e.g. sports clinics or sports introduction days, organized by a sport federation or a community organization). |
| S5. Education-Alcohol-Home Setting | Interventions comprised informative messages distributed via various media channels (e.g. websites, television, newsletters, letters to parents). They aimed to provide knowledge about the harmful effects of alcohol, drugs or smoking, or about newly introduced interventions targeting alcohol, drugs or smoking. The interventions sometimes also included ‘home parties’: incidental, small-scale and easily accessible information sessions for specific target groups (e.g. parents with an immigrant background) conducted at the home setting. |
| S6. Regulation-Alcohol-Commercial Building | Interventions included the implementation of and adherence to national or local regulations (e.g. age legislation for buying alcohol, a breathalyzer test before entering a bar/club, an alcohol ban during children’s activities at sports clubs). These interventions aimed to control the alcohol consumption among young people. |
| S7. Education-Alcohol-Health or Welfare Building | Interventions included a diversity of small-scale, short and easily accessible courses, resilience training courses, consultations or meetings with health professionals. They aimed to provide knowledge and create awareness among parents and young people about the risks of – especially excessive – alcohol consumption. Some also addressed drugs and smoking. The activities or sessions mostly took place in an institute for care and treatment of addiction or in a hospital setting, but sometimes also at a Municipality Health Organization. |
| S8. Facilitation-Overweight-School Setting | Interventions in the school area included offering incidental or permanent small-scale activities to promote physical activity (e.g. school sports days) and providing easily accessible healthy food products (e.g. free fruits or fresh juices in the school canteen). |
| S9. Education-Overweight-Public Building | Interventions included both courses consisting of a series of sessions and incidental and brief information meetings for specific target groups (e.g., parents with a immigrant background). The interventions were provided at public buildings (e.g. library, town hall) that were accessible for free. The aim was to create awareness of healthy eating and physical activity, and to provide both content and how-to knowledge about both these behaviours. |
