# Supplementary material for: Patterns in bottlenecks for implementation of health promotion interventions: a cross-sectional observational study on intervention-context interactions in the Netherlands
Source: Arch Public Health. 2023 Oct 17;81:183. doi: 10.1186/s13690-023-01196-y (PMC10580618; doi:10.1186/s13690-023-01196-y)
Supplement: Supplementary file 2 — Additional file 2: Supplementary file 2. Questionnaire for the survey on conditions for implementation of interventions. [file 13690_2023_1196_MOESM2_ESM.docx]

**Supplementary file 2. Questionnaire for the survey on conditions for implementation of interventions**

| **Indicate to what extent you agree with the following statements:** | **Strongly disagree** |  |  |  | **Strongly agree** |
| --- | --- | --- | --- | --- | --- |
| *i. I (the implementer)* |  |  |  |  |  |
| 1. have sufficient content knowledge about the theme of the intervention |  |  |  |  |  |
| 2. have sufficient skills required for implementation |  |  |  |  |  |
| 3. perceive benefits of the implementation for my job |  |  |  |  |  |
| 4. am enthusiastic and motivated for implementation |  |  |  |  |  |
| 5. am able to implement the intervention |  |  |  |  |  |
|  |  |  |  |  |  |
| *ii. Co-implementer(s)* |  |  |  |  |  |
| 6. have sufficient content knowledge about the theme of the intervention |  |  |  |  |  |
| 7. have sufficient skills required for implementation |  |  |  |  |  |
| 8. perceive benefits of the implementation for their job |  |  |  |  |  |
| 9. are enthusiastic and motivated for implementation |  |  |  |  |  |
| 10. are able to implement the intervention |  |  |  |  |  |
|  |  |  |  |  |  |
| *iii. The intervention* |  |  |  |  |  |
| 11. offers the implementer(s) sufficient personal benefits |  |  |  |  |  |
| 12. fits in with / can be adapted to the daily working routine of the implementer(s) |  |  |  |  |  |
| 13. is easily accessible for the target group |  |  |  |  |  |
| 14. is easy to implement |  |  |  |  |  |
| 15. is complicated due to many implementers/organizations involved^R^ |  |  |  |  |  |
| 16. it is difficult to keep track of its implementation^R^ |  |  |  |  |  |
| 17. can be suitably adapted to the context (e.g. setting, target group, demands of the target group) |  |  |  |  |  |
| 18. fits into an integrated approach to the health problem |  |  |  |  |  |
| 19. the results can easily be made visible |  |  |  |  |  |
| 20. has been certified |  |  |  |  |  |
|  |  |  |  |  |  |
| *iv. My (the implementer’s) organization* |  |  |  |  |  |
| 21. offers me enough time for implementation |  |  |  |  |  |
| 22. offers me enough opportunities to acquire knowledge and skills required for implementation |  |  |  |  |  |
| 23. gives enough support for the health theme |  |  |  |  |  |
| 24. has sufficient financial resources for implementation |  |  |  |  |  |
| 25. the organization’s size complicates implementation^R^ |  |  |  |  |  |
| 26. the organization’s top-down bureaucracy and hierarchy facilitates implementation |  |  |  |  |  |
| 27. clear agreements have been made about my task and responsibility in the implementation |  |  |  |  |  |
| 28. colleagues give me sufficient support for implementation |  |  |  |  |  |
| 29. the management team gives me sufficient support for implementation |  |  |  |  |  |
| 30. the intervention fits the organization’s policy |  |  |  |  |  |
|  |  |  |  |  |  |
| *v. Co-implementing organization(s)* |  |  |  |  |  |
| 31. offer enough time for implementation |  |  |  |  |  |
| 32. offer enough opportunities to acquire knowledge and skills required for implementation |  |  |  |  |  |
| 33. offer enough support for the health theme |  |  |  |  |  |
| 34. have sufficient financial resources for implementation |  |  |  |  |  |
| 35. the organizations’ sizes complicate implementation^R^ |  |  |  |  |  |
| 36. the collaboration with the other co-organizations complicates implementation^R^ |  |  |  |  |  |
| 37. the organizations’ top-down bureaucracy and hierarchy facilitate implementation |  |  |  |  |  |
| 38. clear agreements have been made about everyone’s tasks and responsibilities in implementation |  |  |  |  |  |
| 39. colleagues of the co-implementing organization(s) give me sufficient support for implementation |  |  |  |  |  |
| 40. the management teams of the co-implementing organization(s) give me sufficient support for implementation |  |  |  |  |  |
| 41. the intervention fits the co-implementing organizations’ policies |  |  |  |  |  |
|  |  |  |  |  |  |
| *vi. Context* |  |  |  |  |  |
| 42. there is enough political support for the intervention |  |  |  |  |  |
| 43. the intervention fits the political agenda |  |  |  |  |  |
| 44. sufficient financial resources are provided by the political-administrative level for implementation |  |  |  |  |  |
| 45. the (results of the) intervention offer the political-administrative level opportunities to boost its profile |  |  |  |  |  |
|  |  |  |  |  |  |
| *vii. Implementation strategy* |  |  |  |  |  |
| 46. a clear implementation plan is available |  |  |  |  |  |
| 47. the right materials required for implementation are available |  |  |  |  |  |
|  |  |  |  |  |  |
|  |  |  |  |  |  |
| **List the five most important factors that impacted the implementation of the intervention.**  **Make use of the above statement numbers or write down a factor if it is currently not on the list.** |  |  |  |  |  |
| 1. |  |  |  |  |  |
| 2. |  |  |  |  |  |
| 3. |  |  |  |  |  |
| 4. |  |  |  |  |  |
| 5. |  |  |  |  |  |

R = reversed scoring
