# Supplementary material for: Patterns in bottlenecks for implementation of health promotion interventions: a cross-sectional observational study on intervention-context interactions in the Netherlands
Source: Arch Public Health. 2023 Oct 17;81:183. doi: 10.1186/s13690-023-01196-y (PMC10580618; doi:10.1186/s13690-023-01196-y)
Supplement: Supplementary file 3 — Additional file 3: Supplementary file 3. Flowchart of the response to the survey on conditions for implementation of interventions. [file 13690_2023_1196_MOESM3_ESM.docx]

**Supplementary file 3. Flowchart of the response to the survey on conditions for implementation of interventions**

Non-response by implementers:

44 implementers, responsible for 135 interventions, did not return a questionnaire

Excluded: 46 questionnaires (= interventions) from 17 implementers

- 9: double questionnaires/cases
- 14: intervention was not implemented or missing answers on > 80% of the questionnaire items
- 23: intervention did not target inhabitants

Based on the project leader questionnaire:

In 30 projects, 181 implementers were responsible for 424 interventions. These implementers received a questionnaire on each of their interventions.

120 implementers (66.3%), responsible for 243 interventions (57.3%) in 30 projects, were included

137 implementers returned questionnaires on 289 interventions
